# Genomic, Antimicrobial Resistance, and Public Health Insights into *Enterococcus* spp. from Australian Chickens

**DOI:** 10.1128/JCM.00319-19

**Published:** 2019-07-26

**Authors:** Mark O’Dea, Shafi Sahibzada, David Jordan, Tanya Laird, Terence Lee, Kylie Hewson, Stanley Pang, Rebecca Abraham, Geoffrey W. Coombs, Taha Harris, Anthony Pavic, Sam Abraham

**Affiliations:** a Antimicrobial Resistance and Infectious Diseases Laboratory, Murdoch University, Murdoch, WA, Australia; b New South Wales Department of Primary Industries, Wollongbar, NSW, Australia; c Australian Chicken Meat Federation, North Sydney, NSW, Australia; d PathWest Laboratory Medicine, Fiona Stanley Hospital, Murdoch, WA, Australia; e Birling Avian Laboratories, Bringelly, NSW, Australia; University of Tennessee at Knoxville

**Keywords:** chicken, enterococcus, vancomycin resistance

## Abstract

Due to Australia’s management of antimicrobial use in poultry, particularly the discontinued use of avoparcin for nearly 20 years, it is hypothesized that vancomycin-resistant enterococci associated with human disease are not derived from poultry isolates. This study evaluated antimicrobial resistance (AMR) of five enterococcal species isolated from Australian meat chickens, genomic features of Enterococcus faecium and Enterococcus faecalis, and the phylogenetic relationship of the poultry-derived E. faecium with isolates from human sepsis cases. All enterococcal isolates from chicken ceca were subjected to antimicrobial susceptibility testing. E. faecium and E. faecalis underwent whole-genome sequencing. E. faecium was compared at the core genome level to a collection of human isolates (*n* = 677) obtained from cases of sepsis over a 2-year period spanning 2015 to 2016. Overall, 205 enterococci were isolated consisting of five different species. E. faecium was the most frequently isolated species (37.6%), followed by E. durans (29.7%), E. faecalis (20%), E. hirae (12.2%), and E. gallinarum (0.5%). All isolates were susceptible to vancomycin and gentamicin, while one isolate was linezolid resistant (MIC 16 mg/liter). Core genome analysis of the E. faecium demonstrated two clades consisting predominantly of human or chicken isolates in each clade, with minimal overlap. Principal component analysis for total gene content revealed three clusters comprised of *vanA*-positive, *vanB*-positive, and both *vanA*- and *vanB*-negative E. faecium populations. The results of this study provide strong evidence that Australian chicken E. faecium isolates are unlikely to be precursor strains to the currently circulating vancomycin-resistant strains being isolated in Australian hospitals.

## Abstract (precis)

Due to Australia’s management of antimicrobial use in poultry, particularly the discontinued use of avoparcin for nearly 20 years, it is hypothesized that vancomycin-resistant enterococci associated with human disease are not derived from poultry isolates.

## INTRODUCTION

Enterococci are a ubiquitous component of the commensal microbiota of terrestrial vertebrates. Humans are exposed to enterococci from a number of sources, including other humans, the environment, and foods contaminated with the intestinal microflora of livestock. A characteristic of enterococci that allows them to readily transfer between hosts is their enhanced ability to survive conditions outside hosts that would be fatal to most other vegetative bacteria (1). Consequently, certain species, such as Enterococcus faecalis and E. faecium, are a prominent cause of opportunistic infections in humans causing disease that ranges in severity from mild to fatal (2). In the last two decades, the treatment of enterococcal disease in humans has been complicated by the emergence of antimicrobial-resistant strains. This has led to an increasing tendency for severe forms of multiple resistance and the resultant reliance on “last line of defense” drugs for therapy, including the glycopeptide antimicrobial vancomycin which is categorized by the World Health Organization (WHO) as a “highest priority critically important antimicrobial” (3). Worldwide, vancomycin-resistant E. faecium (VREfm) has emerged as a major nosocomial pathogen of humans (4). Despite studies demonstrating transmission pathways for hospital associated VREfm clones such as ST203, ST796, and ST80 (5), the poultry industry has been examined as a source of antimicrobial-resistant enterococci causing disease in humans, owing to the pervasiveness of chicken meat in the diet of humans, and the widely documented use of antimicrobials within the poultry production cycle.

The emergence of VREfm has been claimed, in part, to be associated with the use of the vancomycin analog avoparcin in chickens as a growth promotant, which occurred in the European Union from 1975 to 1998 and in Australia from 1978 to 2000 (6) but not in the United States (7). It has been demonstrated that after removal of avoparcin from poultry production systems in some countries, VREfm has persisted for extended periods, possibly due to coselection (8–10). It would also be feasible to hypothesize that reverse zoonotic transmission of enterococci from farm workers to poultry could introduce vancomycin resistance elements into the poultry population in a similar manner to that noted for methicillin-resistant Staphylococcus aureus in pigs (11, 12).

However, studies have shown animal and human enterococci isolates often differ in multilocus sequence type (MLST), particularly in the case of E. faecium. As such, it is most likely that animal origin enterococci are not in themselves a direct threat to human hosts but rather the transfer of genetic content between animal and human strains *in vivo* (13, 14). Previous studies have shown transposons containing vancomycin-resistant genes were the likely cause of VREfm identified in poultry farmers and abattoir workers in the Netherlands (15), and the *vanA* gene can be transferred from poultry enterococci to human enterococci *in vivo* (16).

Enterococci possess vast genetic diversity, and some sequence types such as E. faecalis ST16, a hospital-associated clone that is also reported in livestock (17, 18), and the hospital-associated E. faecium ST17 are considered to be more associated with nosocomial infections and carry more resistance genes (19, 20). More recently, grouping of E. faecium isolates has been performed using Bayesian analysis of population structure, with results providing further evidence of hospital-adapted strains (21). Australia is recognized as having a higher proportion of vancomycin resistance among E. faecium isolates collected in humans compared to Europe, with this increasing proportion predominantly due to the preeminence of isolates carrying the *vanB* gene (22, 23). Furthermore, the majority of VREfm are concurrently resistant to other important and critically important antibiotics such as ampicillin, tetracyclines, high-level gentamicin, erythromycin, and nitrofurans, as well as a lower proportion to fluoroquinolones (24, 25).

Little is known about the antimicrobial resistance, distribution and genetic makeup of enterococci of poultry origin in Australia. In 2007 a national government study reported low level *vanC* mediated resistance in <1% of E. faecalis, with no evidence of vancomycin resistance in E. faecium (26). However, samples for this study were collected in 2003, and there has been no structured, nationwide Australian study on VRE carriage in poultry since.

We hypothesize that chicken is not the origin of vancomycin resistance in human E. faecium in Australia due to the exclusion of avoparcin from all food-producing animals in the country. Moreover, the strict regulation of antimicrobials in Australia has also excluded other critically important drugs from being available for use in meat chicken production, including, fluoroquinolones, colistin, ceftiofur, and gentamicin. Therefore, our study aimed to investigate the antimicrobial resistance and genomic characteristics of E. faecium and E. faecalis isolated from the gut of Australian meat chickens at slaughter. Using whole-genome sequencing, we also investigated the evolution and genetic traits of a collection of Australian isolates of E. faecium obtained from cases of sepsis in humans to understand whether E. faecium originating in chicken was a possible cause.

## MATERIALS AND METHODS

### Sample acquisition and processing.

Between June and November 2016, two hundred pooled cecal samples (five cecal samples in each pool) were collected across Australia from meat chickens being processed for human consumption using the approach adopted for surveillance in the United States (27). The samples were part of a nation-wide survey, obtained from 20 processing plants belonging to seven commercial companies that supply over 95% of the Australian market for chicken meat. The number of samples from each plant was proportional to its processing volume. Sampling was carried out by persons suitably trained and experienced in the collection procedure described, with previous experience in specimen collection at slaughter. Only one sample (which constituted ceca from five chickens) was obtained from any single batch being processed on each day of sampling, and samples were shipped overnight on ice-packs to the primary isolation laboratory.

A 10% homogenized solution of cecal samples was prepared in sterile buffered peptone water (Thermo Fisher). The prepared sample was streaked directly onto Bile Esculin Agar (Thermo Fisher), and incubated at 37°C for 48 h. Enterococcal isolates were identified using matrix-assisted laser desorption ionization–time of flight mass spectrometry (Vitek 2 bioMérieux; Bruker Microflex). Once the species was determined, a bacterial colony was plated onto Columbia sheep blood agar (Edwards, Australia) and incubated overnight at 37°C. E. faecium and E. faecalis antimicrobial susceptibility was determined by the broth microdilution method using CMV3AGPF Sensititre National Antimicrobial Resistance Monitoring System (NARMS) panels (Trek Diagnostics, Thermo Fisher Scientific) according to Clinical and Laboratory Standards Institute (CLSI) guidelines adapted for the Sensititre system, with all isolates tested once against each antimicrobial. NARMS breakpoints were used for antimicrobials lacking the CLSI standards (28). The MIC was determined by digital imaging using the Sensititre Vizion system (Trek; Thermo Fisher), and the results were interpreted and verified independently by two laboratory scientists. The antimicrobials and the concentration ranges used are listed according to their antimicrobial class in Table S1 in the supplemental material. Quality control was performed using E. faecalis ATCC 29212 and Staphylococcus aureus ATCC 25923 and 29213. To allow comparability with other studies two susceptibility breakpoints were used: the CLSI breakpoint (28, 29) and the epidemiological cutoff value (ECOFF). The ECOFF values used were as recommended by the European Committee on Antimicrobial Susceptibility Testing (30). Based on the ECOFF value, the isolates were categorized into wild type and non-wild type. Based on the CLSI breakpoint, isolates resistant to at least three antibiotic classes were categorized as multidrug-resistant (MDR).

### Whole-genome sequencing.

Whole-genome sequencing was performed on all E. faecium and E. faecalis isolates. DNA was extracted using the MagMAX multisample DNA extraction kit (Thermo Fisher Scientific) according to the manufacturer’s instructions. DNA library preparations were conducted using an Illumina Nextera XT Library preparation kit, with variation from the manufacturer’s instructions for an increased tagmentation time of 7 min. Library preparations were sequenced on an Illumina Nextseq platform using a midoutput 2 × 150 kit. Genomic data were *de novo* assembled using SPAdes (31). All isolates were analyzed using the Centre for Genomic Epidemiology (CGE; http://www.genomicepidemiology.org/) and the Nullarbor pipeline (v1.20) for determining the multilocus sequence type (MLST) and the presence of antimicrobial resistance and putative virulence genes (based on ≥95% sequence coverage and ≥99% sequence identity) (32). Virulence gene detection was also performed using the ABRicate program (within Nullarbor) with the universal virulence gene database downloaded from the CGE.

E. faecium genomic comparisons were performed against 677 E. faecium isolates typed from human hospital sepsis cases collected by the Australian Group on Antimicrobial Resistance (AGAR) Australian Enterococcus Sepsis Outcome Program (AESOP) over the 2-year period from 2015 to 2016. Phylogenetic trees were constructed based on single nucleotide polymorphisms (SNPs) in the core genome. Genome annotations were performed using Prokka (v1.12) (32), and outputs were processed using Roary (v3.8.0) (33) for core genome determination (with core genes defined as being present in 99 to 100% of isolates based on a 90% BlastP setting), and Gubbins (v2.2.3) (34) for recombination removal and alignment. Maximum-parsimony trees were constructed using MEGAX under default settings with a 1,000-bootstrap test of phylogeny (35). Manual annotation of trees was performed in iTOL (v4.2) (36).

### Statistical analysis.

MIC data for each isolate were downloaded directly from the digital imaging reader software (Thermo Scientific Sensititre SWIN) and processed to obtain MIC tables with exact confidence intervals for proportions derived by the Clopper-Pearson method in Stata version 14.2 (StataCorp LLC, College Station, TX). All chicken and human E. faecium isolates were subjected to principal component analysis (PCA) of binomial variables in R for determination of associations by total gene content (37). The 95% density ellipses were calculated (within R) from the specified correlation matrix (i.e., the first two components) and plotted using GGPlot2.

## RESULTS

Overall, 205 individual isolates were obtained from the 200 pooled cecal samples. At least one isolate was obtained from each pool, with five pools showing mixed colony types from which two isolates were obtained. Isolates identified included E. faecium (37.6%), E. durans (29.7%), E. faecalis (20%), E. hirae (12.2%), and E. gallinarum (0.5%). All sequence data obtained from this study was deposited in the NCBI Sequence Read Archive under BioProject ID PRJNA524396.

### E. faecium.

No isolates were clinically resistant to chloramphenicol, gentamicin, vancomycin or teicoplanin. For the aminoglycosides, two E. faecium isolates were resistant to kanamycin. One E. faecium isolate was linezolid resistant. Although just over half of the E. faecium isolates (54.5%) were non-wild type to ampicillin, only 20.8% were classed as clinically resistant. A large proportion of isolates were resistant to quinupristin-dalfopristin (54.5%). Although no resistance was detected for virginiamycin, 13% of the isolates were classified as non-wild type. MIC distributions based on ECOFF and clinical breakpoints for E. faecium are shown in Table 1 . MDR was found in 23.4% of the isolates, with macrolide, streptogramin, and tetracycline the most frequently identified MDR pattern (11.7%).

**TABLE 1:** Distribution of MICs for Enterococcus faecium (*n* = 77) isolated from Australian meat chickens to 14 antimicrobials^*a*^

Antimicrobial	% of isolates with MIC (mg/liter)													% non-wild type (95% CI)	Clinically resistant (%)
	0.25	0.5	1	2	4	8	16	32	64	128	256	512	1,024		
Ampicillin	9.1	7.8	5.2	9.1	13 |	35.1	14.3	5.2	1.3	0	0	0	0	55.8 (44.1–67.2)	20.8
Chloramphenicol*	0	0	0	0	3.9	75.3	20.8	0	0	0	0	0	0	0.0 (0.0–4.7)	0
Daptomycin	14.3	11.7	5.2	33.8	23.4 |	11.7	0	0	0	0	0	0	0	11.7 (5.5–21.0)	
Erythromycin	35.1	3.9	13	9.1	0 |	3.9	35.1	0	0	0	0	0	0	39.0 (28.0–50.8)	39
Gentamicin*	0	0	0	0	0	0	0	0	0	100	0	0	0		0
Kanamycin*	0	0	0	0	0	0	0	0	0	79.2	14.3	3.9	2.6		2.6
Lincomycin*	0	0	11.7	0	0	2.6	85.7	0	0	0	0	0	0		
Linezolid	0	0	0	55.8	42.9 |	0	1.3	0	0	0	0	0	0	1.3 (0.0–7.0)	1.3
Penicillin (benzyl)	27.3	11.7	7.8	7.8	31.2	3.9	5.2 |	5.2	0	0	0	0	0	5.2 (1.4–12.8)	10.4
Quinupristin-dalfopristin*	0	10.4	6.5	28.6	7.8	15.6	19.5	10.4	1.3	0	0	0	0		54.5
Teicoplanin	98.7	1.3	0	0 |	0	0	0	0	0	0	0	0	0	0.0 (0.0–4.7)	0
Tetracycline	0	0	58.4	1.3	0 |	0	0	3.9	36.4	0	0	0	0	40.3 (29.2–52.1)	40.3
Vancomycin	2.6	53.2	33.8	3.9	6.5 |	0	0	0	0	0	0	0	0	0.0 (0.0–4.7)	0
Virginiamycin	53.2	9.1	5.2	9.1	10.4 |	10.4	1.3	1.3	0	0	0	0	0	13.0 (6.4–22.6)	

a Note that E. faecium is intrinsically resistant to lincomycin. The percentages of isolates classified as non-wild type with corresponding 95% confidence interval (95% CI) and the percentages classified as clinically resistant are shown. For each drug, vertical bars show the positions of the ECOFF values, and shaded areas indicate the range of dilutions evaluated. ECOFF values are not presently available for antimicrobials denoted by an asterisk (*), and blank boxes in the table indicate a lack of relevant breakpoints.

No genes conferring vancomycin resistance were identified. Genes conferring resistance to quinupristin (*ermA*, *ermB*, or *msrC*) and dalfopristin (*vatE*) were detected in 85.7 and 37.7% of isolates, respectively, with 54.5% of isolates phenotypically resistant to the combination. Resistance genes to lincosamides (*ermA*, *ermB*, *lnuB*, *lnuA*, and *lsaA*) were detected in 59.7% of isolates. Genes conferring resistance to aminoglycoside (*aadE*) were present in 7.79% of the isolates. The majority of isolates carried tetracycline resistance genes (61.0%), while low carriage of *dfrG* (2.6%) was noted.

All 77 E. faecium isolates were sequenced, and 45 belonged to 18 known STs, with the most frequent being ST492, ST195, ST241, and ST124 (Table S2).

Only six putative virulence genes were detected in the chicken isolates. In contrast, up to 12 putative virulence genes were detected in the 677 AESOP human isolates. Common putative virulence genes between both groups were biofilm-associated *bopD*, *bsh* (bile salt tolerance), *cpsF* (capsular polysaccharide), and biofilm-associated genes *acm* and *scm* with similar proportions of each gene across the chicken and human isolates (38–40). Genes present in human isolates which were not detected in chicken isolates included the surface adhesion-coding gene *sgrA*, the cell wall surface anchor-encoding gene *ecbA* (41), the fibrinogen binding protein-encoding gene *fss3* (42), the capsular locus genes *cps4B* and *cps4D* (43), and *psaA*, which encodes a metal-binding lipoprotein (44). The *sgrA* gene was present in 12.5% of human isolates, with the remainder of genes present at proportions of 8% or lower.

**FIG 1:**
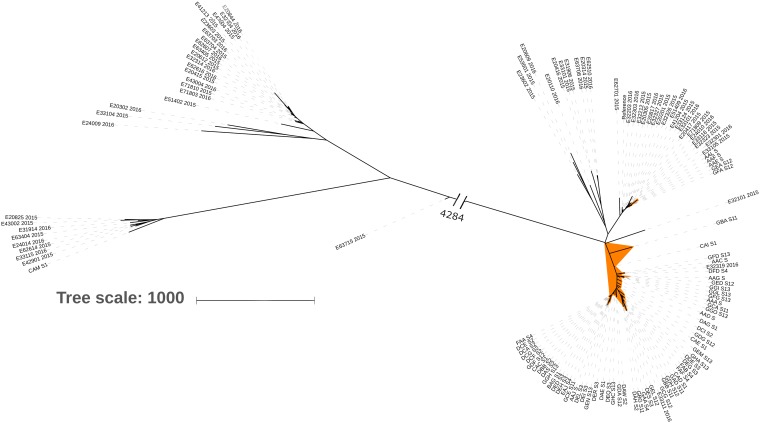
Core genome phylogeny of 69 chicken and 62 human E. faecium isolates displaying five clades consisting of a distinct chicken isolate clade (highlighted orange) and four human isolate clades.

Core genome phylogeny of the chicken and the AESOP human isolates generated three major clades (data not shown), of which two clades were dominated by the respective host species. A third consisted of divergent human isolates (*n* = 29) and a single chicken isolate. All 30 sequences from the divergent cluster, along with a randomly picked subset of the remaining chicken isolate sequences (*n* = 68), and human isolates sitting within the chicken cluster (*n* = 33) were extracted for further comparison. None of these 62 human isolates carried the *vanA* or *vanB* genes. Core genome phylogeny was rederived from this subset, with the isolates distributed into five clades (Fig. 1). Clades 1 and 2 consisted of 30 human isolates and five chicken isolates, and clade 3 consisted of three human isolates and 61 chicken isolates. Clades 4 and 5 were highly divergent from the other three clades, separated by over 4,200 SNPs at the core genome level, and apart from one chicken isolate (clade 4) consisted entirely of human isolates.

**FIG 2:**
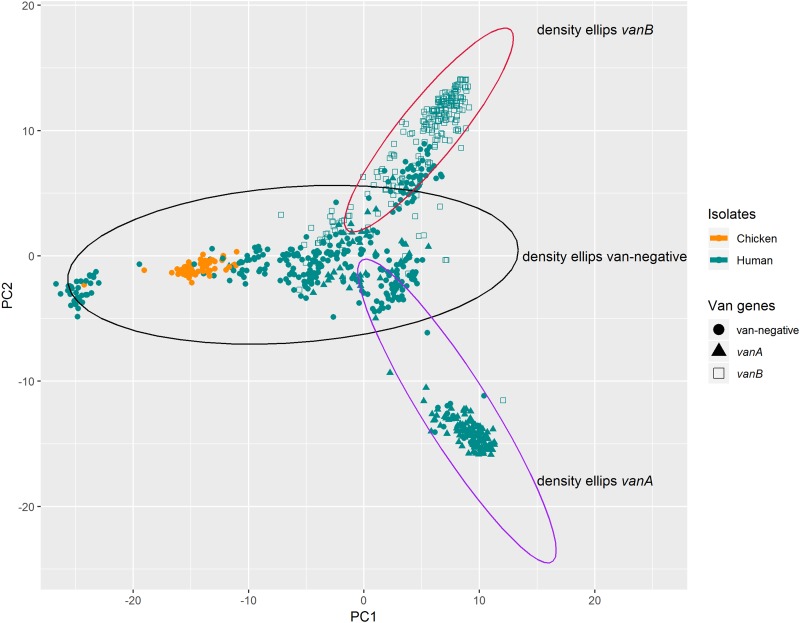
PCA ordination of total gene content for chicken and all human E. faecium isolates (*n* = 677). The 95% density ellipses show three groupings based on the presence of *vanA*, *vanB*, or neither (*van*-negative) genes.

PCA based on total gene content grouped the chicken isolates separately from the human isolates. Based on the presence or absence of the vancomycin-resistant genes (*vanA* and *vanB*), the 95% density ellipses identified three clusters comprised of *vanA*-positive, *vanB*-positive, and *vanA*- and *vanB*-negative populations (Fig. 2). A single chicken isolate, CAM1, associated with a distinct *van*-negative cluster. When this analysis was repeated excluding the presence of *vanA* or *vanB* genes, the clustering effect was unchanged.

### E. faecalis.

All 41 E. faecalis were sequenced, returning 18 known and 4 unknown STs. Eighteen known MLST types were identified with the most prevalent being ST314 (*n* = 7), ST16 (*n* = 5), ST502 (*n* = 4), and ST530 (*n* = 4) (Table S2). No E. faecalis isolates were clinically resistant to chloramphenicol and the aminoglycosides gentamicin and kanamycin. One isolate identified as non-wild type to vancomycin with an ECOFF value of 8 mg/liter. Linezolid resistance was also observed for a single isolate. MIC distributions based on ECOFF and clinical breakpoints for E. faecalis are shown in Table 2. A small proportion of isolates returned MDR (2.4%) phenotypes with a pattern of β-lactam, macrolide, and tetracycline resistance.

**TABLE 2:** Distribution of MICs for Enterococcus faecalis (*n* = 41) isolated from Australian meat chickens to 14 antimicrobials^*a*^

Antimicrobial	% of isolates with MIC (mg/liter)													% non-wild type (95% CI)	Clinically resistant (%)
	0.25	0.5	1	2	4	8	16	32	64	128	256	512	1,024		
Ampicillin	0	2.4	14.6	2.4	61 |	9.8	9.8	0	0	0	0	0	0	19.5 (8.8–34.9)	9.8
Chloramphenicol	0	0	0	2.4	2.4	78	17.1	0	0	0	0	0	0	0.0 (0.0–8.6)	0
Daptomycin	12.2	4.9	12.2	34.1	24.4 |	12.2	0	0	0	0	0	0	0	12.2 (4.1–26.2)	
Erythromycin	48.8	2.4	17.1	4.9	0 |	0	26.8	0	0	0	0	0	0	26.8 (14.2–42.9)	26.8
Gentamicin*	0	0	0	0	0	0	0	0	0	100	0	0	0		0
Kanamycin*	0	0	0	0	0	0	0	0	0	87.8	12.2	0	0		0
Lincomycin*	0	0	4.9	0	0	4.9	90.2	0	0	0	0	0	0		
Linezolid	0	2.4	0	65.9	29.3 |	0	2.4	0	0	0	0	0	0	2.4 (0.1–12.9)	2.4
Penicillin(benzyl)	22	2.4	12.2	9.8	34.1	7.3	2.4 |	9.8	0	0	0	0	0	9.8 (2.7–23.1)	12.2
Quinupristin-dalfopristin*	0	7.3	0	34.1	9.8	22	19.5	7.3	0	0	0	0	0		
Teicoplanin	87.8	9.8	0	0 |	0	0	0	0	0	2.4	0	0	0	2.4 (0.1–12.9)	2.4
Tetracycline	0	0	51.2	0	2.4 |	0	0	7.3	39	0	0	0	0	46.3 (30.7–62.6)	46.3
Vancomycin	7.3	43.9	31.7	12.2	2.4 |	2.4	0	0	0	0	0	0	0	2.4 (0.1–12.9)	0
Virginiamycin	0	0	2.4	17.1	48.8	17.1	7.3	2.4 |	0	4.9	0	0	0	4.9 (0.6–16.5)	

a Note E. faecalis is intrinsically resistant to lincomycin and quinupristin-dalfopristin. The percentages of isolates classified as non-wild type with the corresponding 95% CI and the percentages classified as clinically resistant are shown. For each drug, vertical bars show the positions of the ECOFF values, and shaded areas indicate the ranges of dilutions evaluated. ECOFF values are not presently available for antimicrobials denoted by an asterisk (*), and blank boxes in the table also indicate a lack of relevant breakpoints.

The lincosamide resistance encoding *lsa* gene was detected in 97.6% of isolates. No vancomycin-resistant genes were identified, supporting the phenotypic data. Tetracycline resistance genes (one or more of *tetM*, *tetO*, or *tetL*) were carried in 77.5% of isolates, and 55% of isolates carried the macrolide resistance gene *ermB*. Aminoglycoside-resistant genes included *aadE* (7.3%) and *ant6-la* (4.9%), and the trimethoprim resistance gene (*dfrG*) was present in 4.9% of the isolates.

For all isolates, putative virulence genes included the pilus-encoding genes *ebpB* and *ebpC*, the quorum-sensing gene *fsrB*, the gelatinase production gene *gelE*, and the thiol peroxidase gene *tpx* (45–47). More than 90% of isolates contained pili encoding gene *epbA*, the adhesin encoding gene *efaAfs* , and the collagen binding gene *ace* (46, 48). Twenty-five percent of isolates carried the aggregation-encoding *agg* gene, which was common to all ST16 isolates. The greatest number of putative virulence genes were detected in the single ST100 isolate with the additional carriage of cytolysin genes *cylB*, *cylL*, *cylM*, and *cylA* (49).

### E. durans, E. hirae, and E. gallinarum.

MIC distributions based on ECOFF and clinical breakpoints for *E. durans*, *E. hirae*, and a single *E. gallinarum* combined are shown in Table 3. As for the other enterococcal isolates, no vancomycin resistance was detected, and the resistance profiles were similar to E. faecium and *E. faecalis* with the exception of overall lower ampicillin resistance (1.1%). Eleven percent of the *E. durans* isolates were MDR; the most predominant phenotype was macrolide, tetracycline, and chloramphenicol resistant (6.6%). A low frequency of isolates (1.6%) demonstrated resistance to four antimicrobial classes inclusive of macrolide, phenicol, and tetracycline, with the addition of β-lactam, lincosamide, or fluoroquinolone. Of the *E. hirae* isolates, 20% were MDR, with 16% displaying resistance to macrolide, phenicol, and tetracycline; 4% were resistant to β-lactam, macrolide, and phenicol.

**TABLE 3:** Distribution of MICs for other *Enterococcus* spp. (*n* = 87) comprising Enterococcus hirae (*n* = 25), Enterococcus durans (*n* = 61), and Enterococcus gallinarum (*n* = 1) isolated from Australian meat chickens^*a*^

Antimicrobial	% of isolates with MIC (mg/liter)													% non-wild type (95% CI)	Clinically resistant (%)
	0.25	0.5	1	2	4	8	16	32	64	128	256	512	1,024		
Ampicillin	31	6.9	17.2	16.1	19.5 |	8	0	1.1	0	0	0	0	0	9.2 (4.1–17.3)	1.1
Chloramphenicol*	0	0	0	1.1	6.9	69	23	0	0	0	0	0	0	0.0 (0.0–4.2)	0
Daptomycin	10.3	9.2	11.5	24.1	32.2 |	12.6	0	0	0	0	0	0	0	12.6 (6.5–21.5)	
Erythromycin	35.6	6.9	13.8	9.2 |	0	0	34.5	0	0	0	0	0	0	34.5 (24.6–45.4)	34.5
Gentamicin*	0	0	0	0	0	0	0	0	0	100	0	0	0		0
Kanamycin*	0	0	0	0	0	0	0	0	0	83.9	13.8	2.3	0		0
Lincomycin*	0	0	9.2	1.1	0	0	89.7	0	0	0	0	0	0		
Linezolid	0	1.1	0	59.8	39.1 |	0	0	0	0	0	0	0	0	0.0 (0.0–4.2)	0
Penicillin (benzyl)	17.2	11.5	5.7	20.7	32.2	6.9	1.1 |	4.6	0	0	0	0	0	4.6 (1.3–11.4)	5.7
Quinupristin-dalfopristin*	0	8	4.6	24.1	11.5	20.7	26.4	4.6	0	0	0	0	0		63.2
Teicoplanin	95.4	3.4	1.1	0 |	0	0	0	0	0	0	0	0	0	0.0 (0.0–4.2)	0
Tetracycline	0	0	54	0	1.1 |	0	3.4	3.4	37.9	0	0	0	0	44.8 (34.1–55.9)	44.8
Vancomycin	5.7	46	36.8	8	3.4 |	0	0	0	0	0	0	0	0	0.0 (0.0–4.2)	0
Virginiamycin*	36.8	8	14.9	11.5	8	6.9	6.9	6.9	0	0	0	0	0		

a The percentages of isolates classified as microbiologically resistant with corresponding 95% CI and the percentages classified as clinically resistant are shown. For each drug, vertical bars show the positions of the microbiological breakpoints, and shaded areas indicate the ranges of the dilutions evaluated. Microbiological breakpoints are not presently available for antimicrobials noted by an asterisk (*), and blank boxes in the table also indicate the lack of relevant breakpoints. *E. hirae* breakpoints were used for this table. Note that *Enterococcus* spp. are intrinsically resistant to lincomycin.

## DISCUSSION

In this study of *Enterococcus* spp. from Australian meat chickens, resistance to some antimicrobials of human importance was observed. However, the overall prevalence of antimicrobial resistance was low. No clinical resistance to the critically important antimicrobials gentamicin and vancomycin was detected, and the lack of vancomycin resistance identified in our study provides strong evidence that Australian meat chickens are not responsible for the high rate of vancomycin resistance in E. faecium isolates obtained from Australian hospitals (23, 25). Although avoparcin was extensively used in chicken production in Australia during the 1990s, it was voluntarily withdrawn from the market in 1999, followed by regulatory withdrawal, after concerns that in-feed usage may lead to vancomycin resistance (50). In contrast, recent data from the Australian hospital system shows a high rate of use of vancomycin (22), and perhaps this, combined with the impact of international travel, better explains the extent of VREfm in human isolates in Australia.

Resistance to tetracyclines was frequently identified among all enterococci, potentially due to its occasional use as an in-feed or in-water medication (51). The basis for the high resistance to erythromycin is unclear since macrolides, including erythromycin and tylosin, are rarely used in the industry (52). Although 40% of enterococci in our study were resistant to erythromycin and tetracycline, which are rated as critically and highly important, respectively, for human health according to the WHO (3), this is of less concern to public health, since these drugs are generally not used for the treatment of enterococcal infections in humans.

In Australia, approximately 50% of E. faecium isolated from bacteremia in humans are vancomycin resistant (25). In contrast, we did not identify any VREfm in the chicken ceca sampled. The absence of vancomycin resistance among the poultry isolates indicates human disease-associated VREfm occurrence may be driven by hospital use of glycopeptide or other antimicrobials that select for this resistance. Linezolid is another critically important antibiotic used in human health, and resistance was identified in a single isolate. However, no *cfr* or *optrA* genes were identified, indicating resistance may be due to chromosomal mutations. The most common chromosomal mutation is a G2576T substitution in domain V of the 23S rRNA; however, this was not found in any of the isolates from this study, potentially indicating an undocumented resistance mechanism or overestimation of the MIC (53). Resistance to quinupristin-dalfopristin, which is a highly important antibiotic used to treat VREfm (54) was frequent, with approximately half of the E. faecium isolates being resistant. Whole-genome sequencing demonstrated 37.7% the isolates carried resistance to the quinupristin-dalfopristin combination, with 85.7% of isolates carrying genes for resistance to quinupristin (*ermA*, *ermB*, or *msrC*) and 37.7% for dalfopristin (*vatE*). Although quinupristin-dalfopristin is not used in poultry, resistance is likely driven by the use of virginiamycin (another member of the streptogramin class) or may be due to the historical use of avoparcin in poultry feed (55). Quinupristin-dalfopristin and vancomycin resistance in E. faecium has been reported in countries with a history of avoparcin use in food animals (56, 57). Daptomycin is a critically important antibiotic in treating resistant bacteria in humans. Although it is not used in poultry, 11.7% of E. faecium isolates exceeded the daptomycin ECOFF and were classified as “non-wild type.” However, none were daptomycin resistant according to CLSI interpretive criteria.

Approximately 21% of isolates were ampicillin resistant. No β-lactamases encoding genes were found in our study, indicating resistance is most likely due to mutations within the penicillin-binding protein (*pbp5*) region (58). Examination of the *pbp5* region in all clinically resistant isolates revealed 11 different variants demonstrating common mutations, including A68T (62.5%), M485T (87.5%), N601Y (87.5%), K626E (81.2%), and E629V (81.2%). However, similar proportions of these mutations were also noted in susceptible isolates. In addition, no clinical resistance to gentamicin was observed and, coupled with the low carriage of aminoglycoside-resistant genes (*aadE* and *ant6-la*), suggests that the potential risk of transfer of gentamicin-resistant genetic elements from poultry to human enterococci is very low in Australia. Although resistance to kanamycin and erythromycin were observed, this may be due to the intrinsic resistance of E. faecium to lincosamides and aminoglycosides (59).

Genome analysis of the chicken E. faecium isolates with the AGAR-derived human E. faecium isolates at both the core gene and the pan-gene level demonstrated five distinct groups with minimal overlap. Further analysis of chicken and selected human isolates showing the highest level of genetic relatedness produced five clades. Interestingly, none of the human isolates within this group carried the *vanB* gene. This may indicate that there is a genetic bias toward acquiring vancomycin resistance or that the organisms had no prior exposure to isolates carrying *vanB*. Only three human sepsis-associated isolates clustered with the chicken isolate clade, potentially indicating transfer at some point from chickens to humans. The three sepsis-associated isolates had different STs, consisting of ST12, ST192, and an unknown ST; none of the isolates carried the *vanB* gene. Five ST492 isolates were found in meat chickens and were clustered with the human isolates as opposed to the other chicken isolates. These isolates were also *vanB* negative and may indicate reverse-zoonotic transmission from humans to chickens at some point in the production chain (60).

Pan-genome PCA generally reflected the core genome SNP analysis. Given that this analysis includes combinations of over 16,000 genes, an assessment of the results provides more insight than the core genome analysis alone. The results indicate that particular genome conformations have evolved that are more likely to acquire *van* genes, and this can be further differentiated to include the acquisition of *vanA* or *vanB*. The core genome SNP analysis and the PCA of the pan-genomic analysis indicated that the isolates formed separate clades. In addition, the repeat of the analysis excluding the presence of *vanA* and *vanB* genes demonstrated no change in the clustering, indicating that the gene configuration, rather than the presence or absence of *van* genes, determined the observed clusters.

Although vancomycin susceptible, one E. faecalis isolate was identified as a vancomycin non-wild type with an MIC of 8 mg/liter. A single E. faecalis isolate demonstrated clinical resistance to another glycopeptide, teicoplanin, despite the absence of the *vanA* operon, in which the *vanZ* gene confers resistance (61).

Linezolid clinical resistance at an MIC of >16 mg/liter was also observed for one E. faecium and one E. faecalis isolate. As for E. faecium, none of the isolates from this study carried *cfr* genes, and the single resistant isolate did not carry the G2576T mutation (53).

Ampicillin resistance was present at a lower level in E. faecalis (9.8%) compared to E. faecium (20.8%). While this was not a statistically significant difference between species, it is indicative of a subset of resistant bacteria in Australian poultry, and a focus on measuring this outcome more frequently and accurately is justified. Maintaining susceptibility to ampicillin in both animal and human isolates is a priority because it reduces the need to use antimicrobials of higher importance. However, to detect small changes in the level of ampicillin resistance may require a different approach to surveys because the sample size required is much greater than that used in this and most other similar studies (62, 63).

Despite detecting multiple putative virulence genes in both E. faecium and E. faecalis, these are likely more associated with niche fitness in the poultry host than direct causes of host pathogenicity since they are predominantly associated with binding properties and not strongly associated with pathogenicity in hospital-acquired VRE. Of note was the absence of *esp*, an enterococcal surface protein-coding gene commonly associated with hospital-acquired VRE isolates (64, 65). In addition, the *hyl* gene associated with hospital-acquired VRE and carried on a mobile genetic element (66) was not found in any isolates, and this lack of virulence-associated marker genes provides further evidence that chicken isolates are genotypically separate from hospital-acquired human isolates. Interestingly, the greatest range of putative virulence genes was carried by an E. faecalis ST100, a sequence type that was recently described as a cause of vertebral osteomyelitis lesions in poultry, and it may be that ST100 is potentially virulent in the poultry host compared to the majority of enterococcal strains (67).

In conclusion, our study has provided further insight into the widespread occurrence and characteristics of the potentially pathogenic *Enterococcus* species E. faecalis and E. faecium in Australian meat chickens. Although some enterococcal isolates were found to be resistant to multiple antimicrobials, vancomycin resistance was not detected. The lack of *vanA* and *vanB* carriage in chicken isolates was also associated with core genome and pan-genome separation from human sepsis isolates. When total gene composition was assessed, meat-chicken isolates were in a non-*van* cluster, potentially indicating a genomic type that does not readily acquire vancomycin resistance. This detailed genomic study comparing poultry-derived E. faecium isolates with human sepsis-associated isolates combined with phenotypic antimicrobial resistance data provides evidence that poultry E. faecium is not a primary source of VREfm in Australia.

## Supplementary Material

Supplemental file 1
