# Supplementary material for: Genomic, Antimicrobial Resistance, and Public Health Insights into Enterococcus spp. from Australian Chickens
Source: J Clin Microbiol. 2019 Jul 26;57(8):e00319-19. doi: 10.1128/JCM.00319-19 (PMC6663891; doi:10.1128/JCM.00319-19)
Supplement: Supplemental file 1 [file JCM.00319-19-s0001.pdf]

Table S. 1 Breakpoints used for susceptibility testing of *Enterococcus* spp. isolated from chickens at slaughter in Australia

Class	Agent	Species	Range (mg/L)	ECOFF Breakpoint c	Clinical Breakpoint a b	
					CS	CR
Aminoglycosides	Gentamicin	All	128 - 1024	- d	≤500	>500b
	Kanamycin d	All	128 - 1024	-	≤512	≥1024b
Glycopeptides	Vancomycin	All	0.25 - 32	4	≤4	≥32
	Teicoplanin	All	0.25 - 128	2	≤8	≥32
Lincosamide	Lincomycin d	All	1 - 8	-	≤2	≥8b
Lipopeptides	Daptomycin	All	0.25 - 16	4	≤4	-
Macrolides	Erythromycin	*E. faecium, E. faecalis*	0.25 - 8	4	≤0.5	≥8
		E. hirae	0.25 - 8	2	≤0.5	≥8
Oxazolidinones	Linezolid	All	0.5 - 8	4	≤2	≥8
Penicillins	Ampicillin	All	0.25 - 64	4	≤8	≥16
	Benzylpenicillin	*E. faecium, E. faecalis*	0.25 - 16	16	≤8	≥16
Phenicols	Chloramphenicol	*E. faecium, E. faecalis*	2 - 32	32	≤8	≥32
		E. hirae	2 - 32	8	≤8	≥32
Streptogramins	Quinupristin-Dalfopristin	*E. faecium*	0.5 - 32	-	≤1	≥4
	Virginiamycin	*E. faecium*	0.25 - 128	4	-	-
		E. faecalis	0.25 - 128	32	-	-
		E. hirae	0.25 - 128	-	-	-
Tetracyclines	Tetracycline	All	1 – 32	4	≤4	≥16b

a CLSI VETO1S2 (1) or M100S (2) breakpoints (mg/L), CS = clinically-sensitive; ; CI = clinically-intermediate (between CS and CR, not shown); CR = clinically-resistant

b NARMS (2017) (3) breakpoints (mg/L)

c EUCAST (4) epidemiological cut-off values (mg/L)

d Not defined

Table S. 2. Frequency and proportion of different MLST found in *Enterococcus faecium* and *Enterococcus faecalis* isolates

**Enterococcus faecium**			**Enterococcus faecalis**		
MLST	Frequency	**Percentage**	**MLST**	**Frequency**	**Percentage**
ST492	7	8.97	ST314	7	17.07
ST195	5	6.41	ST16	5	12.20
ST241	5	6.41	ST502	4	9.76
ST124	4	5.13	ST530	4	9.76
ST10	3	3.85	ST202	2	4.88
ST507	3	3.85	ST444	2	4.88
ST517	3	3.85	ST835	2	4.88
ST640	3	3.85	ST22	1	2.44
ST8	2	2.56	ST59	1	2.44
ST236	2	2.56	ST82	1	2.44
ST158	1	1.28	ST100	1	2.44
ST190	1	1.28	ST136	1	2.44
ST194	1	1.28	ST249	1	2.44
ST240	1	1.28	T287	1	2.44
ST245	1	1.28	ST403	1	2.44
ST511	1	1.28	ST477	1	2.44
ST944	1	1.28	ST616	1	2.44
ST1243	2	1.28	ST634	1	2.44
unknown	31	42.31	unknown	4	9.76
**Total**	**77**		**Total**	**41**	

References

1. CLSI. 2015. Performance standards for antimicrobial disk and dilution susceptibility tests for bacteria isolated from animals; 3rd Edition. Clinical and Laboratory Standards Institute, Wayne, PA.

2. CLSI. 2016. Performance standards for antimicrobial susceptibility testing: 26th Edition, vol 35. Clinical and Laboratory Standards Institute, Wayne, PA.

3. NARMS. 2017. National Antimicrobial Resistance Monitoring System: NARMS integrated report, 2015. U.S. Department of Health and Human Services, Laurel, MD.

4. EUCAST. 2016. Breakpoint tables for interpretation of MICs and zone diameters: Version 6.0, valid from 2016-01-01. http://www.eucast.org/fileadmin/src/media/PDFs/EUCAST_files/Breakpoint_tables/v_8.1_Breakpoint_Tables.pdf. Accessed
